# Recurrent Opportunistic Infections in a Thymectomised Patient with Myasthenia Gravis and Good’s Syndrome

**DOI:** 10.7759/cureus.3130

**Published:** 2018-08-10

**Authors:** Nidhi Shankar Kikkeri, Swathi Beladakere Ramaswamy, Sachin M Bhagavan, Raghav Govindarajan

**Affiliations:** 1 Neurology, University of Missouri, Columbia, USA; 2 Neurology, University of Missouri, Columbia , USA

**Keywords:** myasthenia gravis, good’s syndrome, thymoma, histoplasmosis, mycophenolate, immunodeficiency, hypogammaglobulinemia, intravenous immunoglobulins

## Abstract

We report the case of a 65-year-old man with myasthenia gravis, who developed recurrent opportunistic infections following thymectomy and immunosuppressive therapy. Subsequent evaluation including immunological studies, flow cytometry, and bone marrow studies confirmed the diagnosis of Good’s syndrome. The patient was successfully treated with intravenous immunoglobulin (IVIG) and has remained stable with a monthly IVIG regimen. Good’s syndrome should be strongly suspected when patients with myasthenia gravis develop recurrent opportunistic infections, especially after discontinuation of immunosuppressive therapy. Any delay in diagnosis can be life-threatening in such patients. Serum immunoglobulin levels and flow cytometry should be considered part of the initial diagnostic evaluation in patients with myasthenia gravis and an anterior mediastinal mass prior to the initiation of immunosuppressive therapy.

## Introduction

Myasthenia gravis (MG) is a chronic neuromuscular condition, which is considered a prototype of both synaptic and autoimmune disorders. About 60% of thymomas are associated with MG while only 10% of MG patients have thymoma [1-2]. Good’s syndrome (GS), also known as thymoma with immunodeficiency (TWI), is a rare adult-onset immunodeficiency disorder characterized by thymoma and hypogammaglobulinemia, with defects in B and T cell-mediated immunity [2-5]. It was first reported by Robert A Good, who was a physician and scientist at the University of Minnesota Medical School, USA, in the year 1954 [6]. The hallmark features of GS include recurrent sino-pulmonary, opportunistic fungal and viral infections [3, 7]. Thymoma can manifest clinically in the form of autoimmune (30%) diseases or immunodeficiency (6%-11%) disorders [3-4, 8]. The management of the former requires immunosuppression whereas the latter needs immuno-supplementation. Myasthenia gravis has a favorable outcome following thymectomy, whereas patients with Good’s syndrome might worsen following the surgery [8]. The objective of this case report is to establish the rare co-existence of Good’s syndrome in MG patients with post-thymectomy status who present with recurrent opportunistic infections and to demonstrate the role of intravenous immunoglobulins (IVIG) in their treatment and stabilization.

## Case presentation

A 65-year-old male with a past medical history significant for hypertension initially presented with episodes of double vision, fatigue, dysphagia, and generalized weakness. Neurological examination was remarkable for ptosis of the right eye which improved with the ice pack test. Serology was positive for anti-acetylcholine receptor antibodies. Further workup revealed a decremental response to slow (2Hz) repetitive nerve stimulation of the right spinal accessory nerve. The patient was then diagnosed with myasthenia gravis. A computed tomography (CT) scan of the chest revealed thymoma for which the patient underwent resection and was subsequently placed on a high dose (50 mg daily) oral prednisone, in addition to mycophenolate and pyridostigmine. 

Four months after starting the above treatment, the patient presented to the hospital with shortness of breath. A chest X-ray revealed reticulonodular infiltrates. Further workup led to the diagnosis of histoplasmosis. Mycophenolate was then stopped. However, the patient was on a tapering dose of prednisone. The patient then developed refractory diarrhea and was diagnosed with Cytomegalovirus (CMV) colitis. Subsequently, the patient was completely weaned off steroids. However, he continued to develop recurrent pneumococcal infections.

Eight months post discontinuation of steroids, the patient developed disseminated candidal infection. Immunological studies were remarkable for hypogammaglobulinemia (immunoglobulin G (IgG): 100 mg/dl; normal IgG: 700-1600 mg/dl). There was cutaneous anergy to intra-dermal antigen challenge. Subsequently, flow cytometry revealed reduced mature circulating B cells, reduced CD4 count, and reversal of the CD4:CD8 ratio (patient value: 0.5; normal CD4/CD8 ratio: 2.0). The patient then underwent a bone marrow biopsy which revealed reduced pre-B cell lineage. This led to the diagnosis of Good’s syndrome. The patient was successfully treated with IVIG (1g/kg) and since then has remained stable on a monthly IVIG regimen which is used to treat both MG and Good's syndrome.

## Discussion

Patients with myasthenia gravis are commonly treated with corticosteroids, cytotoxic drugs (mycophenolate, azathioprine), alkylating agents (cyclophosphamide), and calcineurin inhibitors (cyclosporine A, tacrolimus) [9]. These immunosuppressive drugs predispose the patients to infections [10]. Hence, during opportunistic infections, the immunosuppressive therapy is gradually weaned off or withdrawn to aid in the recovery from infections [10-11]. Recurrent opportunistic infections after the discontinuation of immunosuppressive therapy should raise the suspicion for an immunodeficiency disorder. 

The incidence of Good’s syndrome is 0.15 cases per 100,000 populations per year, and the average age of the affected patients is 40-70 years [4]. The immunodeficiency spectrum involves both B cells and T cells as reflected by low levels of all types of immunoglobulin, CD4 T-cell lymphopenia, and an abnormal or inverted CD4:CD8+ T-cell ratio [12]. GS is recognized as a distinct entity by the expert committee of the World Health Organization (WHO) / International Union of Immunological Societies on Primary Immunodeficiencies [3]. There are no clear diagnostic criteria for Good’s syndrome. Treatment of Good’s syndrome involves immunoglobulin replacement to maintain adequate trough IgG values [3]. The prognosis of Good’s syndrome appears to be poor. Hermaszewski et al. [13] found that only 33% of patients were alive at the end of 10 years in comparison with 97% of patients with common variable immunodeficiency. 

Thymoma can be associated with both MG and GS. But their concurrent existence in a thymoma patient is a rare association [11]. There have been very few cases reporting their co-existence. A delay in recognizing GS can lead to an infectious catastrophe as seen in our patient. Hence, serum immunoglobulins and flow cytometry should be considered as a part of the initial diagnostic evaluation in patients with MG and an anterior mediastinal mass, prior to the institution of immunosuppressive therapy.

## Conclusions

Good’s syndrome is a rare immunologic disorder characterized by adult onset of immunodeficiency in the setting of thymoma. As it is a rare condition, a high degree of suspicion is needed to diagnose it when a patient with a history of thymectomy presents with recurrent opportunistic infections. The delay in diagnosis can lead to life-threatening infections as seen in our patient. Also, in patients with MG who develop recurrent opportunistic infections with or without immunosuppressive therapy, flow cytometry should be considered to rule out GS. When co-existence of myasthenia gravis and Good's syndrome is confirmed, immediate institution of IVIG therapy is necessary for effective treatment, stabilization, and to prevent the future occurrences of opportunistic infections. 
